# Comparison of the BOPPPS model and traditional instructional approaches in thoracic surgery education

**DOI:** 10.1186/s12909-022-03526-0

**Published:** 2022-06-09

**Authors:** Kang Hu, Rui-Jie Ma, Chao Ma, Qing-Kang Zheng, Zhi-Gang Sun

**Affiliations:** 1 Department of Thoracic Surgery, Jinan Central Hospital, Cheeloo College of Medicine, Shandong University, Jinan, 250013 People’s Republic of China; 2 School of Clinical Medicine, Weifang Medical University, Weifang, 261053 People’s Republic of China; 3 Department of Thoracic Surgery, Central Hospital Affiliated to Shandong First Medical University, Jinan, 250013 People’s Republic of China

**Keywords:** BOPPPS model, Traditional instructional approach, Thoracic surgery education, Clinical practice, Medical education

## Abstract

**Background:**

BOPPPS (bridge-in, learning objective, pretest, participatory learning, posttest, and summary) is a student-centered modular teaching model that improves classroom teaching effectiveness. This study’s primary aim was to explore whether the BOPPPS model has advantages over traditional instructional approaches in teaching lung cancer courses to clinical medical interns.

**Methods:**

A total of 88 students majoring in clinical medicine of Shandong First Medical University and Shandong University, who had clinical practice in thoracic surgery from January 2018 to December 2019, were divided into two groups, receiving the same lung cancer teaching content. The experimental group (*n* = 44) utilized the BOPPPS model, while the control group (*n* = 44) used the traditional instructional approach. A questionnaire was used to attain the students’ satisfaction and self-evaluation of the course, and a post-study examination was used to assess end-of-course performance.

**Results:**

The experimental group’s theoretical examination scores with the BOPPPS teaching model were significantly higher than those in the control group. Students preferred the BOPPPS model more than the traditional instructional approach in course satisfaction, student–teacher interaction, learning initiative, analytical ability, clinical thinking ability, and self-study ability (*p* < 0.05).

**Conclusions:**

Compared with the traditional instructional approach. The BOPPPS model can better inspire clinical medical students’ enthusiasm for thoracic surgery and enhance the students' comprehensive ability. In a word, the BOPPPS model has better teaching effectiveness in the clinical teaching practice of thoracic surgery, which is worthy of reference and popularization.

## Background

Thoracic surgery is an essential branch of surgery. Compared with other branches, thoracic surgery is more challenging, facing more complex diseases, and the patient’s condition is generally aggressive and critical. Therefore, the requirements for doctors’ basic theoretical knowledge and practical ability are very high. At present, clinical medical students have few practical courses in school. There are some problems such as poor operation skills and disconnection between theory and practice after entering clinical practice. It is difficult to improve clinical interns’ professional ability in thoracic surgery in a short period. Modern thoracic surgery has been developing continuously, which puts forward higher medical education requirements [1].

The traditional instructional approach focuses on lecture-based learning as the teaching center, emphasizing syllabus and concept delivery [2, 3]. Although there is no standard definition of “traditional” and much depends on the individual teacher. In China, teachers explain theoretical knowledge, and the students only take notes and accept the knowledge passively [4], while medicine education is complicated and boring [5, 6]. For undergraduate interns, their theoretical courses are mainly learned through the traditional instructional approach. It is difficult to mobilize students’ enthusiasm by using the traditional instructional approach alone in clinical practice [7]. Students’ theoretical knowledge is also relatively insufficient, so it is challenging for them to accept teachers’ content within a limited time [8]. Combined with the actual clinical practice situation in thoracic surgery, the traditional instructional approach is relatively simple, lacks innovation, cannot stimulate interns’ enthusiasm. Hence, it is not easy to achieve exemplary teaching results. Traditional instructional approaches have proved to be not as effective as other teaching strategies in practical application and critical thinking ability [9–11].

The BOPPPS model has initially been proposed by the Center for Teaching and Academic Development, University of British Columbia, Canada [12]. This model emphasizes the student-centered teaching concept and modularizes the classroom teaching process. The BOPPPS model divides the instructional process into six distinct steps: bridge-in, objective, pre-assessment, participatory learning, post-assessment, and summary. Teachers can design instructional content, evaluate and revise the instructional process according to these six steps. Participatory learning is the core part of this teaching model. Teachers can guide students to discuss clinical cases related to the course actively, find problems, and solve problems, effectively improving teaching effectiveness.

BOPPPS model has been used in the practical teaching of many subjects, including dental materials education [13], oral histopathology [14], physiology education [15], healthcare and management education [16]. However, there is no report on the application of BOPPPS model in thoracic surgery education, whether in China or other countries. Although the BOPPPS model are proven to be successful and highly effective at improving the academic knowledge of the students, it is unclear whether the BOPPPS model could work well in thoracic surgery education for the clinical medical student in China. This study selected 88 five-year undergraduate students who completed clinical probation in the Department of Thoracic Surgery of our hospital to conduct the research and preliminarily explored the different effects of the BOPPPS model and traditional methods of thoracic surgery education.

## Methods

### Participants

The study was conducted with 88 students majoring in Shandong First Medical University and Shandong University’s clinical medicine, who had clinical practice in thoracic surgery from January 2018 to December 2019. In 2018, 44 interns in the control group adopted the traditional teaching method. Moreover, in 2019, 44 interns in the experimental group adopted the BOPPPS model. There were 21 females and 23 males in the control group, with an average age of 22.4. The experimental group consisted of 20 males and 24 females, with an average age of 22.5. There was no statistical difference between the two groups in terms of gender, age, entrance achievement, family background, and self-study ability (*p* > 0.05). All the participants signed the informed consent, and the Jinan Central Hospital Ethics Committee approved this study.

### Design

Both groups of students used the same chapter "lung cancer" as the teaching content. All teaching processes were completed within the same time in both the experimental group and the control group. For teaching cases, a lung cancer patient was selected from the inpatients in the department of thoracic surgery. The teacher communicated with patients in advance and obtained their consent, and then the teacher edited the patient-related medical information into a case. The specific teaching methods are as follows.

The control group was mainly taught in the traditional teaching model. The teacher first explained the relevant theoretical knowledge of the selected disease according to the syllabus’s specific requirements. Afterwards, students discussed and answered clinical questions based on the cases provided by the teacher. Finally, the teacher summarized the course content according to the requirements of the syllabus.

**Fig. 1:**
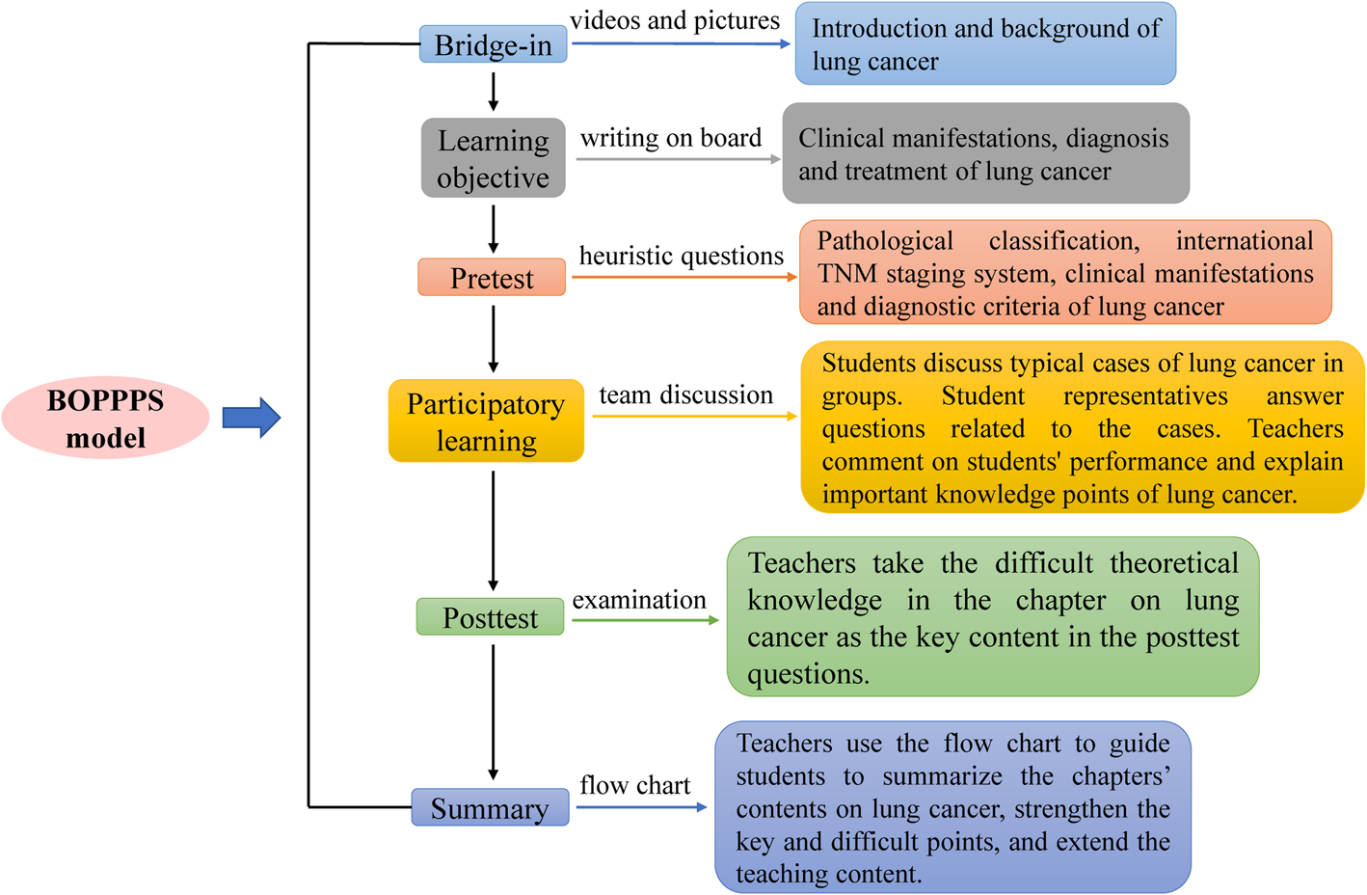
Example of class design for the BOPPPS model

The experimental group used the BOPPPS model to carry out teaching. One week before the internship, the instructor informed the students of the theoretical chapters and related issues. The BOPPPS model was divided into the following six stages. *Bridge-in:* According to the teaching content, the teacher connected the content to be learned with the essential knowledge points such as anatomy, pathophysiology, and diagnostics, leading to the focus and application value of the study from the simple to the deep. *Learning objective:* According to the syllabus, the teacher defined the learning objectives and emphasized the key points and difficulties of teaching. *Pretest:* Lecturing mainly by asking heuristic questions, the clinical teacher could assess students’ level of knowledge, which helped adjust the teaching emphasis in the subsequent teaching. *Participatory learning:* Firstly, the students were divided into groups and selected classic cases according to the discussion’s teaching content. Then, a representative of each group was chosen to answer the questions involved in the cases. Finally, the teacher commented on each group’s answer results and explained the important and difficult points in the cases. Students can consult textbooks and literature, exchange collected information, and have group discussions. They can also analyze and summarize the questions raised and supplement each other to deepen the impression. *Posttest:* Teachers took the difficult theoretical knowledge in the chapter on lung cancer as the key content in the posttest questions. Through the posttest to master the students’ learning effect on the teaching content, teachers can adjust the teaching plan’s difficulty and improve the teaching plan. *Summary:* Teachers used the flow chart to guide the students to sum up the contents of this lecture, strengthened the key and difficult points, and extended the teaching content. Using the lung cancer chapter in this course as an example, the design of a class is shown in Fig. 1.

### Effectiveness assessment

At the end of the course, the two teaching methods’ effectiveness and satisfaction were evaluated in an examination and an anonymous questionnaire.

The theoretical knowledge is in the form of a written examination (a total score of 100 points). The examination questions are randomly selected from the examination question bank, which mainly evaluates the students’ knowledge of lung cancer theory.

Both groups of students participated in the questionnaire survey. Eighty-eight questionnaires were sent out, and eighty-eight were effectively received with an effective recovery rate of 100%. The questionnaire’s content mainly includes course satisfaction (I am satisfied with the design of the course and the way the teachers teach it), student–teacher interaction (I agree with the teacher-student communication method shown in this course), learning initiative (I feel that I can take the initiative to learn in this course), analytical ability (I think my ability to analyze problems can be improved through this course), clinical thinking ability (I think my clinical thinking ability has been significantly improved after class), and self-study ability (my ability to acquire knowledge independently through this course has been improved). (Likert five-level scoring method is used as the evaluation standard, and 1 ~ 5 points means completely dissatisfied ~ completely satisfied).

### Statistical analysis

All statistical analyses were performed using the SPSS version 26.0 software and the Microsoft Office. The measurement data were expressed as mean ± standard deviation (x ± s). Significance was assessed from an independent sample *t*-test. Statistical significance was set as *p* < 0.05.

## Results

### Comparison of test scores between the two groups

**Fig. 2:**
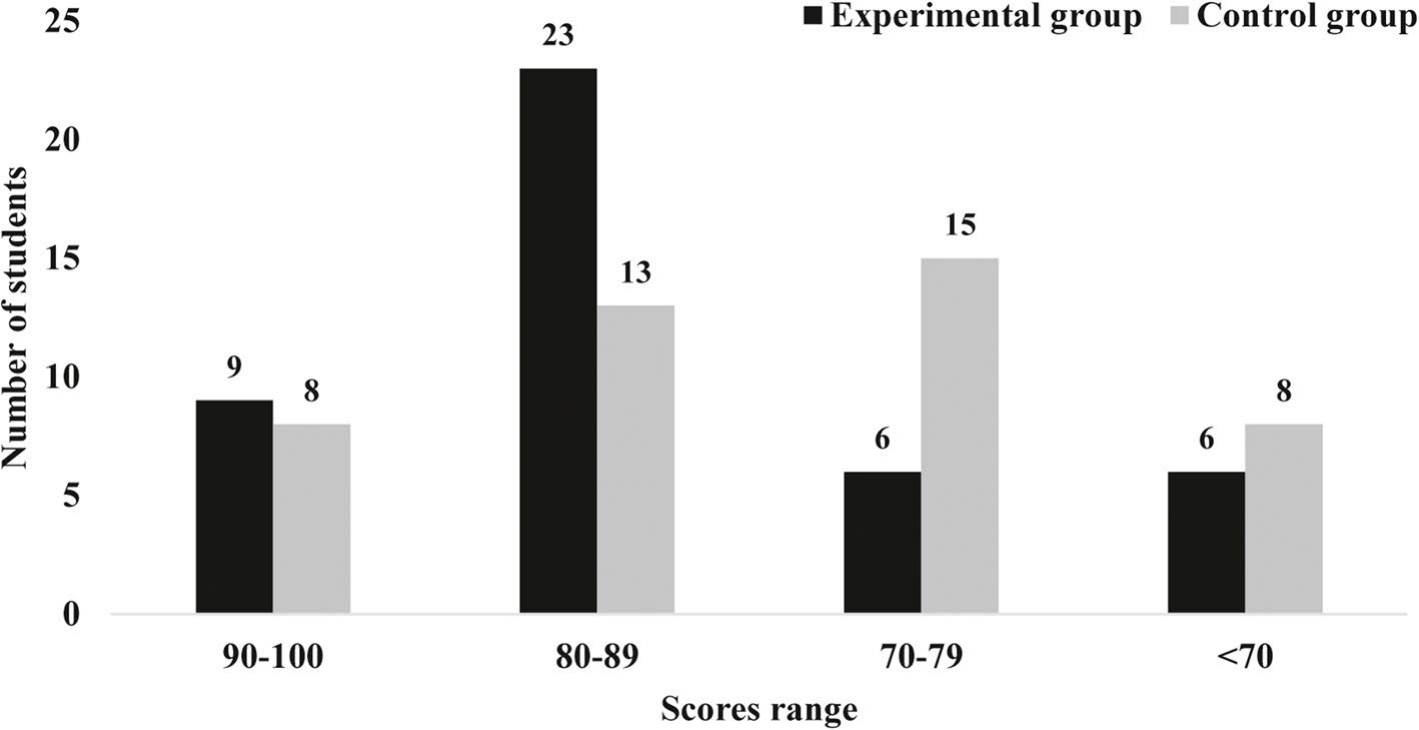
Distribution chart of students’ theoretical test scores

As shown in Table 1 and Fig. 2, the scores of theoretical examinations in the experimental group under the BOPPPS model were significantly higher than those in the control group (81.4773 ± 10.9215 > 76.3636 ± 10.6402), and the difference was statistically significant (*p* < 0.05).

**Table 1:** Comparison of testing scores between experimental and control groups

Group	Control group (*n* = 44)	Experimental group (n = 44)	*t*-value	*P*-value
Final examination scores	76.3636 ± 10.6402	81.4773 ± 10.9215	2.225	0.029

### Comparison of students’ satisfaction and self-evaluation between the two groups

**Fig. 3:**
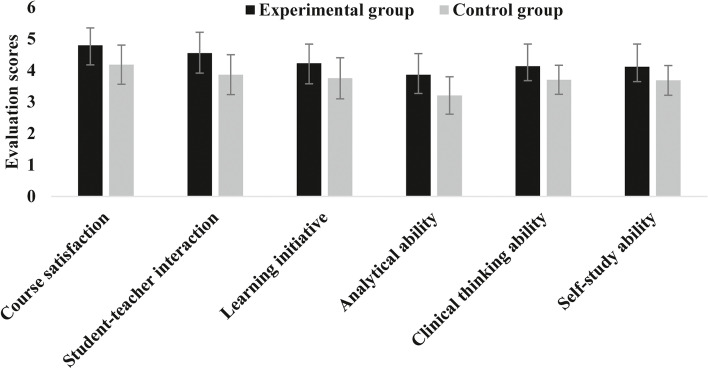
Evaluation of student questionnaire

Students preferred the BOPPPS model (experimental group) more than the traditional teaching methods (control group) in terms of course satisfaction, student–teacher interaction, learning initiative, analytical ability, clinical thinking ability, and self-study ability (Fig. 3). The difference was statistically significant (*p* < 0.05) (Table 2).

**Table 2:** Comprehensive evaluation of two teaching models by two groups of students

Group	Control group (*n* = 44)	Experimental group (*n* = 44)	*t*-value	*P*-value
Course satisfaction	4.1818 ± 0.6203	4.7955 ± 0.5532	4.897	0. 000005
Student–teacher interaction	3.8636 ± 0.6321	4.5455 ± 0.6631	4.937	0.000004
Learning initiative	3.7500 ± 0.6515	4.2273 ± 0.6048	3.561	0.001
Analytical ability	3.2045 ± 0.5938	3.8636 ± 0.6679	4.892	0. 000005
Clinical thinking ability	3.7045 ± 0.4615	4.1364 ± 0.7019	3.410	0.001
Self-study ability	3.6818 ± 0.4712	4.1136 ± 0.7222	3.322	0.001

## Discussion

After completing theoretical courses and entering clinical probation, students majoring in clinical medicine often lack a comprehensive understanding of the process of disease diagnosis and treatment, so it is difficult for them to link the theoretical knowledge with clinical practice fully. Traditional teaching methods are mostly simple teacher-led teaching. The diseases of thoracic surgery are complex, abstract, and difficult to understand, which often leads students to feel dull, lack enthusiasm, and poor learning effect. Therefore, how to stimulate students’ interest in thoracic surgery, improve the clinical teaching effect and optimize the teaching methods are important problems that need to be solved in the current teaching process. The BOPPPS model is a student-centered teaching method and observation system adopted by many famous colleges and universities in Canada in recent years [12, 13]. This teaching method has incomparable advantages over traditional teaching methods in terms of stimulating students’ learning interest and enthusiasm and improving teaching efficiency, which is consistent with this study’s results.

The primary purpose of a clinical internship is to deepen and consolidate the basic knowledge and cultivate the students’ clinical thinking ability so that they can use theoretical knowledge to solve the specific problems encountered in clinical work. The results of this study showed that the BOPPPS model was significantly better than the traditional instructional approach in the aspects of student–teacher interaction, learning initiative, analytical ability, clinical thinking ability, and self-study ability. The experimental group was also significantly better than the control group in the theoretical knowledge examination. Participatory learning is the core part of this teaching model. Teachers can guide students to discuss clinical cases related to the course actively, find problems, and solve problems, effectively improving teaching effectiveness. Additionally, teachers can also obtain feedback information from students in time to adjust subsequent teaching activities. The BOPPPS model can fully inspire students’ enthusiasm for studying and guide them to solve problems on their initiative. Simultaneously, it can also improve students’ clinical thinking ability and cultivate students’ independent learning and communication and cooperation ability. Also, teachers can make continuous progress and improve their teaching methods in teaching activities.

The BOPPPS model is of great help to improve thoracic surgery’s teaching quality, but some problems and puzzles need to be further improved in the subsequent instructional practice. In Fig. 2, we found that there was little difference in the number of students between the experimental group and the control group when the score was above 90 and below 70. We observed that when the scores were between 70–79, the number of students in the control group (15) was more than that of the students in the experimental group (9). On the contrary, in the score range of 80–89, the proportion of students in the experimental group (23) was significantly higher than that in the control group (13), indicating that most of the students in the experimental group were in this score range. We speculated that the BOPPPS model may help improve students whose scores are in the 70–79 range, while it has less effect on students with scores above 90 and below 70. Of course, we need further large sample multicenter studies to confirm this view. All observed differences may also be attributed to the Hawthorne effect. If the effect of the BOPPPS model is indeed caused by the Hawthorne effect, then the BOPPPS model changes students’ behavior rather than ability. The students who participated in the experiment will not improve significantly in other courses and follow-up courses. If BOPPPS can improve students’ ability, then the relatively high scores obtained by students can be reflected not only in this course, but also in the follow-up courses. Therefore, in the next study, we will compare the results of various courses between the experimental group and the control group after the implementation of the BOPPPS model to evaluate whether the improvement of students’ learning effect results from the improvement of learning motivation or the improvement of learning ability. Different from traditional theoretical teaching, the BOPPPS model puts forward higher requirements for the teacher, which requires teachers to change the traditional instructor-led teaching concept. The participation of students increases the difficulty of teaching and requires teachers to have high theoretical learning quality and rich clinical practice experience. In the teaching process, teachers need to effectively guide students to explore and solve problems based on their interests. Also, in the practice of the BOPPPS teaching model, we find that teachers still need to explain the theoretical or abstract teaching contents in detail. Therefore, the BOPPPS model should be applied selectively according to the content of the course and based on the students’ knowledge background. Finally, the BOPPPS model should be an open instructional design model. Teachers should integrate their rich teaching experience into daily instructional practice on the basis of abiding by the BOPPPS teaching model. We should adjust the instructional design according to the instructional content and students’ foundation to make it more in line with students’ psychological characteristics and cognitive laws.

## Conclusion

In the present study, we compared the effects of the BOPPPS model and traditional instructional approaches in thoracic surgery teaching. We found that students’ overall score in traditional courses was lower than those with the BOPPPS model. The BOPPPS model can stimulate students’ interest in thoracic surgery’s clinical probation and improve students’ problem-solving and clinical analysis abilities. Besides, students can also enhance their communication skills with teachers and patients through teamwork, which will lay a good foundation for future clinical work.

Further rigorous large-sample multicenter studies are needed to confirm whether the BOPPPS model is superior to traditional instructional approaches in overall teaching effectiveness. After continuous exploration and ongoing effort, the BOPPPS model will play a more significant role in medical teaching reform to improve teaching effectiveness and quality.

## Data Availability

All data generated or analyzed during this study are included in this published article. The SPSS raw dataset can be provided on request. The corresponding author, Zhi-Gang Sun, will provide additional data, if requested.

## Abbreviation

BOPPPS: bridge-in, learning objective, pretest, participatory learning, posttest, and summary
